# Variable patterns of daily activity participation across settings in autistic youth: A latent profile transition analysis

**DOI:** 10.1177/13623613231154729

**Published:** 2023-02-28

**Authors:** Yun-Ju Chen, Eric Duku, Anat Zaidman-Zait, Peter Szatmari, Isabel M Smith, Wendy J Ungar, Lonnie Zwaigenbaum, Tracy Vaillancourt, Connor Kerns, Teresa Bennett, Mayada Elsabbagh, Ann Thompson, Stelios Georgiades

**Affiliations:** 1 McMaster University, Canada; 2 Tel Aviv University, Israel; 3 The Hospital for Sick Children, Canada; 4 University of Toronto, Canada; 5 Centre for Addiction and Mental Health, Canada; 6 Dalhousie University, Canada; 7 University of Alberta, Canada; 8 University of Ottawa, Canada; 9 The University of British Columbia, Canada; 10 McGill University, Canada

**Keywords:** autism, environment, latent transition, participation profiles, youth

## Abstract

Participation in daily activities is often linked to functional independence and well-being, yet individual variability in participation and factors associated with that variation have rarely been examined among autistic youth. We applied latent profile analysis to identify subgroups of youth based on parent-reported activity participation frequency at home, school and community, as well as associations with youth characteristics, family demographics and environmental supportiveness among 158 autistic youth (aged 11–14 years at baseline). Three-, three- and two-profile solutions were selected for home, school and community settings, respectively; the most prevalent profiles were characterized by frequent home participation (73%), low participation in non-classroom activities at school (65%) and low community participation, particularly in social gatherings (80%), indicating participation imbalance across settings. More active participation profiles were generally associated with greater environmental support, higher cognitive and adaptive functioning and less externalizing behaviour. Latent transition analysis revealed overall 75% stability in profile membership over approximately 1 year, with a different home participation profile emerging at the second time-point. Our findings highlighted the variable participation patterns among autistic youth as associated with individual, family and environmental factors, thus stressing the need for optimizing person–environment fit through tailored supports to promote autistic youth’s participation across settings.

**Lay abstract:**

What people do or engage in in their daily lives, or daily life participation, is often linked to their state of being happy and healthy, as well as potential for living independently. To date, little research has been conducted on daily activity participation by autistic youth at home, at school or in the community. Learning more about individual differences in participation levels and what might influence them can help to create custom supports for autistic youth and their families. In this study, 158 caregivers of autistic youth were asked how often their children took part in 25 common activities at two assessments, about one year apart. The analysis showed three profiles for each of the home and school settings and two profiles for the community setting. These profiles reflected distinct patterns in how often autistic youth took part in various daily activities, particularly in doing homework, school club activities and community gatherings. Most autistic youth were in profiles marked by often taking part at home but less often at school and in the community, and about three-fourths of them tended to stay in the same profile over time. Autistic youth with limited participation profiles were more likely to have lower scores on measures of cognitive ability and daily life skills and more challenging behaviour, and faced more barriers in their environment. These findings show how important it is to think about each autistic person’s strengths and weaknesses, and changing needs, to better support their daily life participation.

## Introduction

Adolescence is a time of great physical and psychological growth and change, which often poses increased demands on autistic youth than on their neurotypical peers (Cheak-Zamora et al., 2017; Wehman et al., 2014). For children and youth with and without autism or other developmental challenges, active participation in everyday life situations or daily activities (e.g. helping with household chores, participating in sports with peers, grocery shopping) is often linked to positive development towards functional independence and better quality of life (Folta et al., 2022; World Health Organization, 2001; Yeung & Towers, 2014). Regular involvement in a variety of daily activities also has the potential to ameliorate the impact of perceived stress on satisfaction with life and to facilitate socio-emotional adjustment (Bishop-Fitzpatrick et al., 2017; Bohnert et al., 2019; Howells et al., 2019). The extent to which autistic youth integrate into their communities through daily activity participation reflects their strengths and needs and can be an important indicator of successful transition into adulthood (Clark & Adams, 2020; Lounds Taylor et al., 2017; Myers et al., 2015; Weiss et al., 2021).

Previous researchers have reported on autistic youth’s limited participation in daily activities across different settings (Lamash et al., 2020; Tint et al., 2017). For instance, autistic youth may spend more time on solitary activities involving screen-based technology, such as watching TV and using computers, compared to their non-autistic peers (Folta et al., 2022; Kuo et al., 2014; Mazurek et al., 2012; Mazurek & Wenstrup, 2013). They also tend to participate less frequently in social activities with lower engagement in school and community settings (Chang et al., 2019; DaWalt et al., 2019; Lounds Taylor et al., 2017; Shattuck et al., 2011), although this may depend on who (e.g. closeness in relationship and shared identity) is involved in the activities and the level of support for social inclusion (Locke et al., 2010; Orsmond & Kuo, 2011). Prior cross-sectional findings have shown that such disparity between autistic and non-autistic individuals in activity participation seems to be more evident between ages 11 and 17 years than at younger ages (Ratcliff et al., 2018), highlighting the importance of further examination of activity participation among autistic youth.

To date, there remains a paucity of evidence regarding activity participation patterns among autistic youth, with more attention directed to younger children than adolescents (Askari et al., 2015; Lamash et al., 2020). Existing evidence tends to focus on group comparisons (autistic vs non-autistic) for narrowly defined categories of activities (e.g. social media use, leisure activities) in particular settings (e.g. social or community participation). This limits an understanding of individual differences in a broader range of activity participation among the heterogeneous autistic population. Nevertheless, previous association findings have revealed several potential explanatory factors of individual differences in participation among autistic populations. Clinical characteristics, including maladaptive behaviour, cognitive ability and social communication skills, have been observed to be associated with different levels of participation among autistic children and youth (Mazurek et al., 2012; Myers et al., 2015; Shattuck et al., 2011). Aside from individual characteristics, previous research has also shown associations between autistic youth’s activity participation and family socioeconomic status (SES), particularly household income and caregiver education (Budavari et al., 2021; Liptak et al., 2011; Myers et al., 2015; Orsmond & Kuo, 2011; Shattuck et al., 2011). Environmental factors, such as demands or considerations of performing certain activities (e.g. available space, transportation, safety, sensory overwhelmingness) and access to support and resources, were also found to affect autistic youth’s participation (Egilson et al., 2018; Krieger et al., 2018; Lamash et al., 2020). A recent study examined community participation patterns among autistic adults and found that about one-third participated more actively in a variety of activities, whereas others showed more limited participation and experienced more barriers to accessing resources despite motivation to participate (Song et al., 2021). These findings demonstrate that individual, family and environmental factors are associated with levels of participation across settings among autistic populations. Thus, it is important to examine the joint contributions of these factors to autistic youth’s participation to enable tailored opportunities and supports that optimize person–environment fit and facilitate adult competencies (Budavari et al., 2021; Lai et al., 2020).

Another gap in understanding autistic youth’s activity participation is the lack of information concerning changes in participation levels and patterns over time. A previous study of autistic children reported stable participation in home activities but reduced participation in social and physical activities at school as children transitioned into adolescence (Simpson et al., 2019). In another recent study in which a large group of autistic youth and adults was tracked over 3 years (Weiss et al., 2021), a decline in participation in structured weekday activities for at least 25% of those who were recruited during the transition period was observed. This resonates with another longitudinal finding that about half of the autistic young adults no longer engaged in education- or work-related activities following high school exit (Taylor & DaWalt, 2017). These findings indicate that change in activity participation is not only about the *quantitative* degree of participation but also could lie in *qualitative* patterns or profiles of participation in certain types of activities or in particular settings. Further evidence of changes in participation patterns may have important implications for understanding risk and resilience in autism as well as for providing timely support for changing needs across developmental stages (Lai et al., 2020; Leebens & Williamson, 2017).

To address the gaps regarding individual variability and potential changes in activity participation in autistic youth, we aimed to address the following research questions by examining participation patterns or profiles among a cohort of autistic youth from early (ages 11–14 years) to middle adolescence (ages 14–16 years):

*RQ1.* How many distinct profiles (or subgroups) can be identified based on the frequency of participation in types of activities for each of home, school and community settings?
*RQ2.* Are family SES (household income and caregiver education), environmental support/barriers, youth’s cognitive ability and core autism features associated with profile membership?
*RQ3.* Do participants classified by these profiles differ in their levels of adaptive functioning, internalizing/externalizing behaviour and involvement during participation?
*RQ4.* Do participation patterns and profile membership remain stable across assessments at 11–14 and 14–16 years of age?

It was expected that multiple profiles of activity participation would be identified for each setting, with profiles characterized by higher levels of participation generally associated with higher family SES, greater environmental supportiveness and higher functional abilities. However, these associations may vary across settings (e.g. those who only engage in at-home activities may not necessarily have higher functional abilities). We also expected instability in patterns and profile membership over time, particularly for the school and community settings, which are often associated with more social and environmental change and unpredictability (Adreon & Stella, 2001; Kersten et al., 2020).

## Method

### Participants and procedures

The current sample was drawn from the *Pathways in Autism Spectrum Disorder* study, a longitudinal project tracking the developmental trajectories of an inception cohort (*N* = 421) of children diagnosed with autism between 2 and 5 years of age at five sites across Canada (see Table S1 for a comparison of demographic characteristics between the current and full samples). Participating families with informed consent were followed up with repeated standardized assessments and proxy report measures across childhood and adolescence. The study project (see Szatmari et al., 2015 for details) was approved by the research ethics boards at all participating sites. This study focused on two assessments (12.60 ± 3.29 months apart) administered between 2017 and 2019 when youth were 11–14 years (T1; *N* = 158) and 14–16 years of age (T2; *N* = 134).

### Measures

#### Activity participation frequency, involvement and environmental support

The Participation and Environment Measure for Children and Youth (PEM-CY; Coster et al., 2010) is a parent-report questionnaire that evaluates participation in 25 types of daily activities and associated environmental support/barriers across home (10 activities), school (5 activities) and community (10 activities) for children and youth aged 5–17 years. Caregivers were asked to rate their children’s participation frequency (0 = never to 7 = daily) and level of involvement (1 = minimally involved to 5 = very involved in terms of a child’s level of attention, emotional engagement or satisfaction) for each activity over the last 4 months. The PEM-CY includes a separate scale for evaluating environmental supportiveness for the child’s participation in each setting (e.g. to what extent the social, cognitive, physical and sensory demands of activities and access to services hinder or support the child’s participation; 0 = usually makes harder/does not help to 2 = usually helps) for each setting. In this study, PEM-CY data were collected at T1 and T2.

Given a lack of evidence on the psychometric properties of PEM-CY for autistic youth, we examined internal consistency by calculating Cronbach’s alpha for the ‘frequency’, ‘involvement’ and ‘environment’ scales for each setting and time-point. The results are shown in Table S2. The internal consistency of the involvement and environment scales (α = 0.76–0.87) is overall higher than those of the frequency scales (α = 0.52–0.68), thus supporting the use of composite scores for involvement and environmental supportiveness in the analyses, which were calculated by averaging item responses (reverse-coded when appropriate) for each setting. The low internal consistency of frequency scales reflects potential variability across items (i.e. types of activities), thus meriting further latent profile analysis (LPA) using raw item responses of the frequency scale as indicators for parsing such variability within each setting.

#### Family demographics

Information about families’ annual household income and the primary caregiver’s highest level of education was gathered at T1 (or the nearest time-point available) via the Family Background Information Questionnaire (FBIQ) developed for the *Pathways in ASD* project based on questions derived from the Canadian National Longitudinal Study of Children and Youth (Statistics Canada, 1995). The household income (11 ordinal categories ranging from less than CAN$5000 to more than CAN$80,000) and primary caregiver education variables (1 = high school or less; 2 = some post-secondary education; 3 = bachelor’s degree or higher) were used in the current analysis.

#### Core autism features

The Autism Diagnostic Observation Schedule (ADOS; Lord et al., 2000) was administered and scored by research-reliable examiners. The total Calibrated Severity Score (CSS) at the latest time-point available before T1 (between ages 10 and 12 years) was used in the analyses.

#### Cognitive ability (non-verbal intelligence)

For verbal participants (*n* = 127), the non-verbal intelligence quotient (NVIQ) was measured by the Wechsler Abbreviated Scale of Intelligence, Second Edition (WASI-II; Wechsler, 2011), Perceptual Reasoning Index at T1. For other participants with minimal verbal language (*n* = 22), NVIQ was measured with the Leiter-R (Roid & Miller, 1997) or Leiter-3 (Roid et al., 2013), which do not require verbal instructions or responses. These measures are commonly applied in studies with autistic individuals with a wide range of verbal abilities (Plesa Skwerer et al., 2019; Schwartz et al., 2020).

#### Adaptive functioning

The Vineland Adaptive Behavior Scales, Second Edition (VABS II; Sparrow et al., 2005), is a semi-structured parent interview that assesses children’s adaptive behaviour in the domains of Communication (COM), Socialization (SOC), Daily Living Skills (DLS) and Motor Skills. VABS data were collected at T1, and standard scores (M = 100, SD = 15) for the COM, SOC and DLS domains were used in the analyses.

#### Emotional and behavioural concerns

The Child Behavior Checklist 6–18 (CBCL) (Achenbach & Rescorla, 2001) is a norm-referenced instrument for caregivers that assesses children’s emotional and behavioural concerns over the last 6 months. CBCL data were collected at T1, and the total *T*-scores (M = 50, SD = 10) for the Internalizing (including Social Withdrawal, Anxiety/Depression subscales) and Externalizing (including Rule-Breaking and Aggressive Behaviour subscales) behaviour composite scales were used in the analysis.

### Data analyses

#### LPA of activity participation at T1

LPA was leveraged to identify profiles of activity participation frequency with PEM-CY data collected at T1. To avoid model identification issues that may arise from a large number of indicators/parameters, given the relatively small sample size (Collins & Lanza, 2010), all the analyses were performed separately for each setting (i.e. home, school and community). Given that extreme outlier indicators can bias the LPA solutions, we first examined multivariate outliers for the indicators (i.e. frequency for each activity) using the Mahalanobis distance approach with a value of *p* = 0.001 as a cut-off (Spurk et al., 2020). We excluded four and five cases for the home participation frequency scale at T1 and T2, respectively, as well as one case for the T1 community participation frequency scale. LPA models were then fitted with various numbers of profiles. The optimal profile solution was determined based on several model fit indices, including Akaike information criterion (AIC), Bayesian information criterion (BIC), sample size-adjusted Bayesian information criterion (SABIC), entropy and adjusted Lo–Mendell–Rubin likelihood ratio test (LMR-LRT). Specifically, lower AIC/BIC values indicate improved model fit, whereas entropy values closer to 1 reflect more distinct profiles. The LMR-LRT compared fit statistics between neighbouring models, with values of *p* < 0.05 indicating significant improvement in the model fit by adding each additional profile (Tein et al., 2013).

Upon deciding the optimal solution for each setting, each participant was assigned to a profile with the highest posterior probabilities of profile membership. We then examined the associations between T1 profile membership and other variables of interest, which were available at T1 (but not at T2). Vermunt’s (2010) three-step approach was applied to examine the covariate effects of T1 household income, primary caregiver education, environmental supportiveness, NVIQ and core autism features on profile membership (controlling for site). Sex assigned at birth and primary caregiver’s ethnicity were excluded due to the small number of girls (*n* = 22, 14%) and non-White caregivers (*n* = 41, 26%) in our sample, respectively. Each covariate was initially examined individually as a correlate of profile membership before creating adjusted models that included relevant covariates. Next, the Bolck–Croon–Hagenaars (BCH) three-step approach (Bakk & Vermunt, 2016) was used to examine differences across profiles in the level of adaptive functioning (VABS COM, SOC, DLS domain scores), maladaptive behaviour (CBCL Internalizing and Externalizing subscale scores) and involvement during participation across activities. Both three-step approaches account for classification uncertainty by either treating the assigned profile or class as a nominal latent profile indicator (Vermunt’s approach; recommended for evaluating latent profile/class predictors or covariates) or computing weights to reflect probabilities of correct or incorrect classification to each profile (BCH approach; recommended for evaluating distal/concurrent outcomes) for deriving bias-adjusted estimates.

All the latent profile and transition analyses were performed with maximum likelihood robust (MLR) estimation using Mplus 8.7 (Muthén & Muthén, 1998–2017). Cross-tabulation of T1 profile membership across settings was explored with odds ratio (OR) tests.

#### Latent transition analysis of activity participation from T1 to T2

Before conducting latent transition analysis (LTA), LPA was repeated with PEM-CY data collected at T2 to explore the optimal profile solution of activity participation frequency. We then extended the cross-sectional LPAs to an LTA by allowing for a direct effect between the latent profile factors of T1 and T2 for each setting separately. We tested whether the profile structures were stable across T1 and T2 by using LRTs to compare a full measurement non-invariance (or freely estimated) model with a full measurement invariance model in which the same profile structures across time-points were assumed, and a partial measurement invariance model in which some profiles were allowed to be freely estimated across time. If the LRT statistics indicated no significant difference between the complex (full non-invariance) and parsimonious (full or partial invariance) models, the more parsimonious one would be selected (Ryoo et al., 2018). Aside from the evaluation of model parsimony, we also examined the model fit statistics (BIC and entropy) to determine the final LTA model that best describes the observed data. Full information maximum likelihood estimation was used to handle missingness at either time-point so that all information available could contribute to estimating the time-specific parameters, but only cases with complete data at both time-points (*N* = 106) contributed to the estimation of the transition parameters.

#### Community involvement

The overall objectives of the *Pathways in ASD* study were determined by meetings of parents, self-advocates, practitioners and researchers. Community members have been engaged in aspects of the study design over the years since 2005 but not in the formulation of the research questions addressed in this article.

## Results

### Participant characteristics

The included participants had mean ages of 13.87 ± 0.84 and 15.22 ± 0.57 years at T1 and T2, respectively. The distributions of youth’s sex, household income, caregiver’s education and race/ethnicity did not differ between samples across the two time-points, but attrition at T2 was higher at the Edmonton site (see Table 1 for the sample demographics). Descriptive statistics for the numeric variables are presented in Table S3.

**Table 1.** Sample characteristics.

	T1 (*N* = 158)	T2 (*N* = 134)
Sex assigned at birth (% male)	136 (86%)	117 (87%)
Age in years (mean (SD))	13.9 (0.8)	15.2 (0.6)
Site		
Halifax	17 (11%)	17 (13%)
Montreal	62 (39%)	66 (49%)
Hamilton	20 (13%)	18 (13%)
Vancouver	35 (22%)	32 (24%)
Edmonton	24 (15%)	1 (1%)
Household income^a^		
<CAN$40,000	27 (17%)	20 (15%)
CAN$40,000–80,000	45 (28%)	42 (31%)
⩾CAN$80,000	86 (54%)	71 (53%)
Primary caregiver’s education^a^		
High school or less	13 (8%)	10 (7%)
Some post-secondary education	65 (41%)	56 (42%)
Bachelor’s degree or higher	80 (51%)	66 (49%)
Primary caregiver’s ethnicity^a^		
White	111 (70%)	98 (73%)
Other	41 (26%)	36 (27%)

a Reported at T1.

### Latent profiles of activity participation at T1

**Figure 1.**
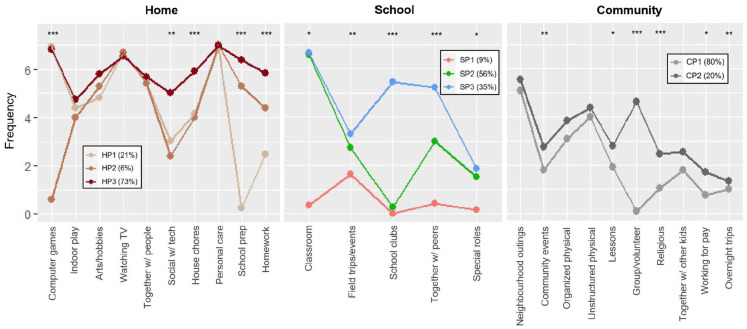
Visualization of participation profiles for each setting at T1 (ages 11–14 years). Kruskal–Wallis tests for differences across latent profiles (^*^p < 0.05, ^**^p < 0.01, ^***^p < 0.001); pairwise comparison results are presented in Table S5. Frequency was measured at an 8-point ordinal scale (0 = never, 1 = once in last 4 months, 2 = few times in last 4 months, 3 = once a month, 4 = few times a month, 5 = once a week, 6 = few times a week, 7 = daily).

Based on the relatively improved BIC/AIC and LMR-LRT results across solutions (see Table S4) as well as levels of distinction between the extracted profiles, the selected profile solutions for each setting are visualized in Figure 1 and described below (detailed latent profile description and comparison results are shown in Table S5). The high entropy values (0.99–1.00) indicated good confidence in group classification under these solutions.

Home participation (HP) profiles (three-class solution) at T1: The three profiles differed significantly in frequency across 5 of the 10 types of activities – school preparation (χ^2^(2) = 86.00, *p* < 0.001), computer games (χ^2^(2) = 59.56, *p* < 0.001), homework (χ^2^(2) = 27.24, *p* < 0.001), household chores (χ^2^(2) = 25.14, *p* < 0.001) and socializing using technology (χ^2^(2) = 12.77, *p* = 0.002).

- HP1 (*n* = 32; 21%): less frequent participation (than weekly basis) in school preparation activities, socializing using technology and household chores;
- HP2 (*n* = 10; 6%): less frequent participation (than weekly basis) in computer games, socializing using technology and household chores;
- HP3 (*n* = 112; 73%): overall frequent participation (on weekly basis) across home activities.

School participation (SP) profiles (three-class solution) at T1: Significant profile differences were observed across all school activities – school clubs (χ^2^(2) = 127.53, *p* < 0.001), classroom (χ^2^(2) = 53.12, *p* < 0.001), together with peers (χ^2^(2) = 32.87, *p* < 0.001), field trips/events (χ^2^ (2) = 10.94, *p* = 0.004) and special roles (χ^2^(2) = 6.47, *p* = 0.039).

- SP1 (*n* = 14; 9%): overall low participation (less frequent than monthly basis) across school activities;
- SP2 (*n* = 87; 56%): less frequent participation (than monthly basis) in non-classroom activities;
- SP3 (*n* = 54; 35%): more frequent participation across school activities, particularly distinct from other profiles in those that involve socializing (e.g. school clubs, together with peers).

Community participation (CP) profiles (two-class solution) at T1: Significant profile differences were observed in group/volunteer activities (χ^2^(1) = 132.35, *p* < 0.001), religious activities (χ^2^(1) = 12.41, *p* < 0.001), community events (χ^2^(1) = 8.99, *p* = 0.003), overnight trips (χ^2^(1) = 6.95, *p* = 0.008), lessons (χ^2^(1) = 5.15, *p* = 0.023) and working for pay (χ^2^(1) = 5.10, *p* = 0.024).

- CP1 (*n* = 125; 80%): overall low participation (less frequent than monthly basis) across community activities, except for neighbourhood outings and physical activities;
- CP2 (*n* = 32; 20%): more frequent participation particularly in community social gatherings (e.g. group/volunteer and religious activities).

### Cross-tabulation of T1 profile memberships across settings

OR tests indicated a significant association between HP and SP profile memberships (χ^2^(4) = 33.28, *p* < 0.001). No significant association was found between SP and CP (χ^2^(2) = 5.28, *p* = 0.071), and between HP and CP profile memberships (χ^2^(2) = 0.03, *p* = 0.99). Specific salient patterns across settings are as follows:

- Youth in the active HP and CP profiles (i.e. HP3 and CP2) were more likely to be in the active SP profile (i.e. SP3) (ORs with 95% confidence intervals = 3.22 [1.24, 8.37] and 2.28 [1.03, 5.05], *p* = 0.016 and 0.043).
- Those in the lower HP profiles (i.e. HP1 and HP2) were more likely to be in the profile of low non-classroom school activity participation (i.e. SP2) (OR = 2.74 [1.18, 6.36], *p* = 0.019).
- Those in the lower SP profiles (i.e. SP1 and SP2) were more likely to be in the low CP profile (i.e. CP1) (OR = 2.28 [1.03, 5.05], *p* = 0.043) and the HP profile characterized by less frequent school preparation (i.e. HP1) (OR = 11.03 [2.52, 48.32], *p* = 0.001) (see Figure S1).

### Covariates of T1 profile memberships

The multinomial logistic regression results (see Table 2) revealed that at T1, youth who experienced higher environmental supportiveness and had higher NVIQ were more likelyto be in the more active participation profiles. Household income was only positively associated with HP (*b_HP3vs1_* = 0.19, SE = 0.07, *p* = 0.007), while the primary caregiver education level was positively associated with HP and SP (*b* = 0.71–1.11, SE = 0.31–0.50, all *p* < 0.05). Youth with milder autism features were more likely to be in the more active SP profile (*b_SP3vs2_* = −0.23, SE = 0.08, *p* = 0.003). After adjusting for the effects of other covariates, environmental supportiveness continued to be a significant covariate across settings (*b* = 1.77–5.33, SE = 0.76–1.52, all *p* < 0.05). NVIQ remained significant for home (*b_HP3vs1_* = 0.02, SE = 0.01, *p* = 0.01) and school settings (*b_SP3vs2_* = 0.03, SE = 0.01, *p* = 0.007), while caregiver education only remained significant in the school setting (*b_SP2vs1_* = 1.61, SE = 0.66, *p* = 0.015 and *b_SP3vs1_* = 1.70, SE = 0.70, *p* = 0.015).

**Table 2.** Covariate effects on participation profile membership at T1 (ages 11–14 years), controlling for site.

T1 predictors	Multinomial logistic regression results, *b* (SE)						
	Home			School			Community
	HP2 vs 1	HP3 vs 1	HP3 vs 2	SP2 vs 1	SP3 vs 1	SP3 vs 2	CP2 vs 1
Household income	0.16 (0.15)	0.19 (0.07)^**^	0.03 (0.15)	0.08 (0.09)	0.22 (0.12)	0.14 (0.09)	0.05 (0.09)
Primary caregiver’s education	0.68 (0.63)	0.71 (0.31)^*^	0.03 (0.58)	**1.05 (0.47)** ^*^	**1.13 (0.50)** ^*^	0.08 (0.29)	0.56 (0.33)
Environmental supportiveness	**2.71 (1.20)** ^*^	**2.68 (0.80)** ^**^	−0.03 (1.06)	**2.57 (1.19)** ^*^	**4.43 (1.21)** ^***^	**1.85 (0.68)** ^**^	**2.01 (0.76)** ^**^
Non-verbal IQ	0.00 (0.02)	0.03 (0.01)^**^	0.03 (0.02)	0.02 (0.01)	0.05 (0.02)^**^	0.03 (0.01)^***^	0.01 (0.01)^*^
Core autism features (ADOS-CSS)	−0.09 (0.13)	−0.10 (0.09)	−0.01 (0.12)	0.02 (0.14)	−0.21 (0.14)	−0.23 (0.08)^**^	−0.08 (0.09)

HP: home participation; SP: school participation; CP: community participation; ADOS-CSS: Autism Diagnostic Observation Schedule-Calibrated Severity Score.

Alternative parameterization of multinomial logistic regression with different reference profiles was performed for obtaining pairwise group comparison results. Bolded beta coefficient values represent significant effects (*p* < 0.05) in the adjusted model.

* *p* < 0.05; **p < 0.01; ***p < 0.001.

### Associated characteristics of T1 profiles

As shown in Table 3, youth in the three HP profiles differed significantly in externalizing behaviour (χ^2^(2) = 6.71, *p* = 0.035), adaptive functioning domains (χ^2^(2) = 32.03–63.88, all *p* < 0.001) and involvement during participation (χ^2^(2) = 18.72, *p* < 0.001). The three SP profiles differed across externalizing behaviour (χ^2^(2) = 14.05, *p* = 0.001), all adaptive functioning domains (χ^2^(2) = 61.28–86.42, all *p* < 0.001) and involvement (χ^2^(2) = 24.82, *p* < 0.001). As for CP, the two profiles differed in externalizing behaviour (χ^2^(1) = 4.91, *p* = 0.027), all adaptive functioning domains (χ^2^(1) = 7.23–10.19, all *p* < 0.01) and involvement (χ^2^(1) = 5.82, *p* = 0.016). Overall, participants in HP3, SP3 and CP2 tended to be more involved in the activities in which they participated, and had higher levels of adaptive functioning and lower levels of externalizing behaviour than those in other profiles.

**Table 3.** Maladaptive/adaptive behaviour and participation involvement by T1 profile (mean estimates (SE)).

Home	HP1 (*n* = 32)	HP2 (*n* = 10)	HP3 (*n* = 112)	Comparisons χ^2^	
				Overall (*df* = 2)	Pairwise
CBCL					
Internalizing Behaviour	59.10 (1.53)	59.10 (2.27)	56.40 (0.99)	2.81	–
Externalizing Behaviour	54.94 (1.57)	55.70 (2.54)	50.90 (0.97)	6.71^*^	1 > 3 (4.81^*^)
VABS					
Communication	58.62 (2.46)	69.20 (6.67)	74.74 (1.43)	32.03^***^	3 > 1 (31.98^***^)
Daily Living Skills	56.28 (1.95)	63.30 (3.37)	74.65 (1.30)	63.88^***^	3 > 1 (61.34^***^); 3 > 2 (9.84^**^)
Socialization	55.00 (2.18)	58.00 (4.31)	73.92 (1.59)	53.57^***^	3 > 1 (49.26^***^); 3 > 2 (12.01^**^)
PEM-CY					
Involvement	3.44 (0.10)	3.68 (0.25)	3.94 (0.05)	18.72^***^	3 > 1 (18.37^***^)
School	SP1 (*n* = 14)	SP2 (*n* = 87)	SP3 (*n* = 54)	Comparisons χ^2^	
				Overall (*df* = 2)	Pairwise
CBCL					
Internalizing Behaviour	58.29 (2.16)	57.85 (1.05)	56.55 (1.52)	0.63	–
Externalizing Behaviour	53.93 (1.96)	54.38 (0.96)	48.07 (1.41)	14.05^**^	1 > 3 (5.91^*^); 2 > 3 (13.49^***^)
VABS					
Communication	56.42 (3.23)	66.26 (1.73)	81.30 (1.70)	63.68^***^	2 > 1 (7.23^**^); 3 > 1 (46.52^***^); 3 > 2 (37.89^***^)
Daily Living Skills	58.49 (2.24)	66.10 (1.67)	78.61 (1.58)	61.28^***^	2 > 1 (7.41^**^); 3 > 1 (53.96^***^); 3 > 2 (29.20^***^)
Socialization	52.34 (2.95)	63.17 (1.73)	81.54 (1.89)	86.42^***^	2 > 1 (10.02^**^); 3 > 1 (63.40^***^); 3 > 2 (50.66^***^)
PEM-CY					
Involvement	2.93 (0.28)	3.53 (0.10)	4.10 (0.10)	24.82^***^	2 > 1 (4.12^*^); 3 > 1 (15.48^***^); 3 > 2 (15.16^***^)
Community	CP1 (*n* = 125)	CP2 (*n* = 32)		Comparisons χ^2^	
				Overall (*df* = 1)	Pairwise
CBCL					
Internalizing Behaviour	57.80 (0.91)	55.34 (1.58)		1.81	–
Externalizing Behaviour	52.88 (0.89)	48.84 (1.59)		4.91^*^	1 > 2
VABS					
Communication	68.74 (1.50)	78.06 (2.63)		9.52^**^	2 > 1
Daily Living Skills	68.22 (1.36)	75.72 (2.43)		7.23^**^	2 > 1
Socialization	67.02 (1.63)	74.35 (2.67)		10.19^**^	2 > 1
PEM-CY					
Involvement	3.63 (0.07)	3.99 (0.13)		5.82^*^	2 > 1

BCH: Bolck–Croon–Hagenaars; HP: home participation; SP: school participation; CP: community participation; CBCL: Child Behavior Checklist; VABS: Vineland Adaptive Behavior Scales; PEM-CY: Participation and Environment Measure for Children and Youth.

* *p* < 0.05; **p < 0.01; ***p < 0.001 (BCH three-step multinomial logistic regression tests).

### Latent profile transitions from T1 to T2

Table S6 displays the fit statistics of LTA models with various degrees of constraints, based on which the best fitting parsimonious solution for each setting was selected. For HP, the LTA model with partial invariance was selected based on the non-significant LRT result, larger entropy values and relatively lower BIC compared with the fully invariant model. Fully invariant LTA models were selected for SP and CP, given the same considerations. The final LTA solutions for each setting were visualized in Figure S2. Next, transition probabilities were calculated to evaluate stability in latent profile membership over time. As shown in Table 4, those who were classified into the most prevalent profiles tended to stay in the same profile over time (e.g. 88.9% of HP3 at T1 stayed in HP3 at T2, and 88.3% of CP1 at T1 stayed in CP1 at T2), whereas a wide range of transition probabilities was observed between the less prevalent profiles. Among the 106 participants who had PEM-CY data at both time-points, 76%, 72% and 78% stayed in the same HP, SP and CP profiles from T1 to T2, respectively, indicating overall 75% within-person stability in profile membership. Those who changed their profile membership (i.e. the ‘movers’) did not significantly differ from their stable counterparts across settings in intervals between the two assessments, T1 household income, caregiver education, environmental supportiveness or autism features, except that the movers in the home setting (60% moved from HP1/2 to HP3; see Table S7) tended to have lower NVIQ (*F*(1,93) = 9.91, *p* = 0.002).

**Table 4.** Latent transition probabilities from T1 (ages 11–14 years) to T2 (ages 14–16 years).

Home			
T2 T1	HP1	HP2^a^	HP3
HP1	**46.2%**	6.0%	47.7%
HP2	0.0%	**0%**	100%
HP3	6.5%	4.6%	**88.9%**
School			
T2 T1	SP1	SP2	SP3
SP1	**13.9%**	69.9%	16.2%
SP2	10.3%	**76.3%**	13.4%
SP3	0.0%	27.4%	**72.6%**
Community			
T2 T1	CP1	CP2	
CP1	**88.3%**	11.7%	
CP2	54.1%	**45.9%**	

HP: home participation; SP: school participation; CP: community participation.

Bolded values on the diagonal indicate transition probabilities to the same profile (i.e. stability). Specific transition patterns of ‘movers’ for each setting can be found in Table S7.

a HP2 at T2 was a different profile from HP2 at T1.

## Discussion

In this study, we provide a comprehensive characterization of daily activity participation patterns among autistic youth from a national inception cohort. Latent profile analyses revealed the existence of heterogeneous subgroups defined by the frequency of participation in a variety of activities across home, school and community settings. Whereas the most prevalent profile in the home setting was characterized by frequent participation overall, the dominant profiles in school and community settings reflected lower participation levels. Across settings, only a small portion of autistic youth (8%) were consistently classified to the highest participation profile across settings (i.e. HP3, SP3 and CP2), while the others showed variations in their participation levels across settings. These indicate participation imbalance across settings among autistic youth, supporting previous evidence that autistic youth tend to spend more time in solitary activities at home than engaging in a broader range of activities beyond the home setting (Lamash et al., 2020; Mazurek & Wenstrup, 2013; Orsmond & Kuo, 2011).

At T1, the distinct profiles were associated with levels of adaptive functioning and externalizing behaviour. Importantly, environmental supportiveness seemed to influence youth’s participation patterns more than did family income, parental education, NVIQ and core autism features, thus reinforcing previous evidence on the impact of environmental support/barriers on autistic youth’s participation (Egilson et al., 2018; Krieger et al., 2018; Lamash et al., 2020). Our finding of low associations between core autism features, cognitive ability and activity participation is not surprising, given the previous evidence of reducing links between these domains as autistic individuals transition into adolescence and adulthood (Duncan & Bishop, 2015; Kanne et al., 2011), which may point to opportunities to promote participation across the clinical continuum. We also found that internalizing behaviour did not differ across participation profiles despite the overall elevated level of internalizing behaviour observed across our sample, which is consistent with a previous observation of autistic children and youth (Dovgan & Mazurek, 2019). Further, the LTA revealed overall stability of profile membership across adolescence for the majority (~75%); among the ‘movers’, those in the lower home and school participation profiles at T1 seemed to shift to a higher participation profile at T2, indicating potential opportunities for increasing participation in these settings among subgroups of autistic youth.

### Home participation

Consistent with previous findings (Folta et al., 2022; Mazurek et al., 2012; Mazurek & Wenstrup, 2013), socializing using technology, such as social media, seemed to be one of the home activities in which autistic youth participated least, given an average frequency of a few times a month in contrast to daily participation in more solitary screen-based activities such as watching TV and playing computer games. Another infrequent home-based activity was indoor play (e.g. playing toys or board games), which might be developmentally appropriate for younger children. Aside from common daily activities such as using computers and personal care, recreational activities (e.g. watching TV, hobbies), helping with household chores and getting together with people were the more frequent home activities among autistic youth (more than once a week on average), but the frequency of participating in many of these activities may still be lower than that seen in non-autistic peers (almost daily) as reported in previous studies (Egilson et al., 2018; Fogel et al., 2021). When examining individual variability by separating our participants into subgroups, we observed that about 73% of our sample participated in a variety of home activities on a daily or weekly basis. The remaining 27% of our sample was less active in home-based activities such as using technology for leisure or social purposes, helping with household chores, school preparation and doing homework, and on average had lower adaptive skills. In addition, the profile characterized by less frequent school preparation and homework (i.e. HP1) was associated with more environmental barriers, which may relate to the cognitive demands of completing schoolwork at home that often require parent involvement (Egilson et al., 2018; Tamm et al., 2020). Our examination of profile membership across settings also highlights the concurrent challenges in school preparation at home and participation in school activities. Thus, individualized support targeting environmental barriers and caregiver problem-solving is needed for at least a subpopulation of autistic youth to ensure consistency in school-related activity participation across settings (Hampshire et al., 2014).

Our LTA revealed that home participation patterns may change across adolescence, with the profile characterized by infrequent computer use at T1 replaced by a profile characterized by limited self-care activities at T2. The transition probability between HP2 at T1 and T2 was zero, indicating that these profiles comprised distinct groups of participants. Although this finding may reflect the instability of the very small profiles, which require future validation with a larger replication sample, the different profile identified at T2 may be due to the increased computer use (Engelhardt & Mazurek, 2014) and difficulties with functional self-care skills over the course of adolescence, with the latter associated with less efficient transition planning and lower expectations for independent living (Reyes et al., 2021). Another interesting finding is that those who changed their profile membership over time in the home setting tended to have lower NVIQ, with 60% of them shifting to the most active home participation profile at T2 (while being mostly stable in other settings). This indicates that at least a subgroup of less cognitively able autistic youth may hold potential for increased participation in home activities such as doing homework and school preparation across adolescence. Further research is merited to identify the factors associated with changes in home participation among autistic youth; the importance of such evidence is underscored by the COVID-19 pandemic (which occurred after the current data collection), during which more frequent in-home activity participation and associated challenges were reported (Garcia et al., 2021).

### School participation

Among our sample, most participants attended classroom activities almost daily, while participation in other types of school activities was limited to a few times per month on average. Our finding is consistent with the previous report that autistic youth tended to participate less in school clubs and socialization with peers, compared to their non-autistic peers’ daily or weekly participation in these activities (Fogel et al., 2021; Lamash et al., 2020). When examining variability within our autistic cohort, we identified three profiles that differed in the frequency of participation, particularly in social activities. About 35% of our sample showed more active social participation at school, such as attending school clubs and spending time with peers. The remaining 65% were classified into profiles characterized by lower school participation (i.e. SP1 and SP2), which were generally associated with more externalizing behaviour, lower adaptive functioning, lower engagement during participation and more environmental barriers. Large differences across participation profiles were observed in all adaptive functioning subdomains, indicating that these skills are highly associated with active school participation (Hastings et al., 2020; Lyons et al., 2016). In addition, caregivers’ education was positively associated with their children’s school participation, which was the only family SES variable that survived after controlling for other covariates. This aligns with a previous finding that higher maternal education was associated with autistic children’s more participation beyond home settings (Little et al., 2014). Such association may be explained by the positive effect of parent involvement and collaboration with teachers or school-based professionals on autistic students’ school performance (Rispoli et al., 2019; Schultz et al., 2016), although the moderating role of caregivers’ backgrounds, including education, merits further research.

Compared to other settings, family and environmental factors were more robust covariates of school participation, indicating that tailored support for both autistic individuals and their families that address environmental barriers may be particularly critical for improving school participation (Kientz & Dunn, 2012). The transition analysis revealed an 86% probability of shifting to a more active school participation profile for those in SP1 (the lowest participation profile) at baseline, indicating that it may take some time for autistic students to become engaged in a variety of school activities. This may also reflect increasing opportunities or support as they progressed into higher school years. However, the overall low participation in non-classroom activities, especially those that involve socialization, may stress the need for additional support to broaden autistic youth’s participation in the school setting, such as providing a more inclusive and interest-based setting (Chen et al., 2021; Koegel et al., 2012) along with adequate environmental adaptations to mitigate barriers during participation (Krieger et al., 2018).

### Community participation

Consistent with previous findings (Budavari et al., 2021; Egilson et al., 2017; Lamash et al., 2020), overall community participation of our participants was observed to be limited (on average, only a few times over several months), except for neighbourhood outings which tended to occur weekly. This may be due to autistic individuals’ generally limited peer relationships that support participation in a variety of events in communities (Dovgan & Mazurek, 2019; Orsmond et al., 2004; Song et al., 2022) and their preference to perform activities alone or with caregivers rather than in a social group (Egilson et al., 2017; Kersten et al., 2020). However, individual variability was observed across activities, particularly in group/volunteer activities and social/religious gatherings. More active participation in these activities was associated with higher adaptive functioning, less externalizing behaviour and fewer environmental barriers. These associations indicate that interventions targeting these areas may help improve autistic youth’s social participation in the community (Carter et al., 2013; Myers et al., 2015), while it is important to recognize that enhancing participation opportunities may in turn have a positive impact on adaptive outcomes (Howells et al., 2019; Orsmond & Kuo, 2011). Our finding of overall stable low community participation across adolescence further reinforces the need for continuous supports in community settings for autistic youth. It is also noteworthy that the low agreement in participation levels between community and other settings may reflect the challenge of generalizing skills that autistic individuals acquired at home or school to community settings, which tend to be less predictable and require higher skill levels (Budavari et al., 2021; Kersten et al., 2020). As community participation is closely linked to independent living and successful transitions into adulthood, interventions at both the individual level (e.g. skill-based training tailored to individual strengths and interests) and societal level (e.g. community-based support systems and accommodations) would be important for supporting autistic youth to thrive across various settings (Budavari et al., 2021; Carter et al., 2013).

### Limitations and future directions

A major limitation of this study is the relatively small sample, which resulted from attrition since study entry. Although we ensured that the smallest extracted profiles represent no less than 5% of the total sample, some of the small subgroups may be unstable or unable to be replicated, and may be underpowered for detecting significant differences in external variables across profiles. Also, the results regarding longitudinal stability may be biased to reflect those who stayed in the study. However, our findings did demonstrate meaningful distinctions between extracted profiles not only in latent profile indicators but also in a range of external variables (i.e. showing criterion validity evidence). Replications with larger samples representing gender and ethnic diversity while controlling for confounding factors such as school type (mainstream vs special education), residential area (rural vs urban) and other family variables (e.g. religious affiliation, families’ social networks) would help better characterize heterogeneous participation patterns.

Another caveat is that, due to the study design and limited sample size, we were not able to test more complex hypotheses, such as identifying the covariates of profile transition probabilities via conditional LTA and examining how profile transitions are linked to changes in associated variables (e.g. household income, environmental supportiveness and involvement). Although this study represents a rare endeavour by tracking autistic individuals’ participation patterns over time, long-term follow-up to adulthood is needed to understand changing daily participation during transitions and its linkage to adulthood outcomes. Further, our reliance on parental reports of youth’s activity participation may be biased by parents’ interpretations and expectations, particularly concerning autistic youth’s involvement during participation, the environmental support/barriers that they experienced, as well as the activities they found meaningful (Willis et al., 2017). Many of our correlate variables also relied on a single informant, so that the magnitude of association may be overestimated. Future research will benefit from the perspectives of autistic individuals and other stakeholders for a more comprehensive understanding of youth’s needs and strengths in daily activity participation.

### Implications

Given that past investigations of the participation of autistic children and youth often focused on participation in specific settings and/or mean-level comparisons with non-autistic peers, this study endeavoured to capitalize on the heterogeneity seen in autism and to characterize patterns of individual differences among autistic youth in daily activity participation. A better understanding of such individual variability is crucial for identifying the strengths, needs and interests of each autistic adolescent, thus facilitating the design and delivery of tailored services and supports. Adding to prior evidence that autistic youth tended to participate more in home-based activities and have limited participation at school and community, we found that individual variability in participation across settings is associated with adaptive functioning and externalizing behaviour, which could be the focus of intervention for subgroups of autistic youth with limited participation; enhancing participation opportunities in various types of daily activities may also in turn facilitate adaptive outcomes. Further, our findings underscore the importance of a supportive environment that provides autistic youth with a wider variety of options for participation, which is a key to social inclusion in communities for autistic individuals (United Nations, 2006; Weaver et al., 2021). Our longitudinal analyses also demonstrate that the participation patterns were generally stable from early to middle adolescence but could change among subgroups. Particularly, autistic youth’s community participation remained stably low or tended to decrease over time. As active participation in the community is often linked to positive adulthood outcomes (Budavari et al., 2021; Song et al., 2021), monitoring autistic youth’s community participation over time can inform tailored and timely intervention according to individual needs in specific settings for promoting a smoother transition into adulthood.

## Supplemental Material

sj-docx-1-aut-10.1177_13623613231154729 – Supplemental material for Variable patterns of daily activity participation across settings in autistic youth: A latent profile transition analysis Supplemental material, sj-docx-1-aut-10.1177_13623613231154729 for Variable patterns of daily activity participation across settings in autistic youth: A latent profile transition analysis by Yun-Ju Chen, Eric Duku, Anat Zaidman-Zait, Peter Szatmari, Isabel M Smith, Wendy J Ungar, Lonnie Zwaigenbaum, Tracy Vaillancourt, Connor Kerns, Teresa Bennett, Mayada Elsabbagh, Ann Thompson and Stelios Georgiades in Autism
