# Supplementary material for: Variable patterns of daily activity participation across settings in autistic youth: A latent profile transition analysis
Source: Autism. 2023 Feb 28;27(8):2241–55. doi: 10.1177/13623613231154729 (PMC10576904; doi:10.1177/13623613231154729)
Supplement: sj-docx-1-aut-10.1177_13623613231154729 – Supplemental material for Variable patterns of daily activity participation across settings in autistic youth: A latent profile transition analysis [file sj-docx-1-aut-10.1177_13623613231154729.docx]

**Supplementary Materials**

Table S1. Demographic characteristics of the current sample (with data collected during adolescence; N=186) and *Pathways in ASD* full sample (N=421)

|  | Current sample  (N=186) | Full sample^†^  (N=421) | Odds ratio  (Current vs. Full) |
| --- | --- | --- | --- |
| Sex assigned at birth (% male) | 160 (86%) | 356 (85%) | 1.12 (.64) |
| Age of diagnosis in months [mean (SD)] | 38.8 (8.5) | 38.2 (8.7) | t(605) = .78 |
| Site | | |  |
| *Halifax* | 26 (14%) | 56 (13%) | 1.06 |
| *Montreal* | 75 (40%) | 135 (32%) | 1.43 |
| *Hamilton* | 24 (13%) | 68 (16%) | .76 |
| *Vancouver* | 37 (20%) | 93 (22%) | .80 |
| *Edmonton* | 24 (13%) | 69 (17%) | .76 |
| Household income | | |  |
| *Less than $40,000* | 33 (18%) | 71 (17%) | 1.06 |
| *$40,000-$80,000* | 57 (31%) | 107 (25%) | 1.30 |
| *$80,000 or more* | 95 (51%) | 211 (50%) | 1.03 |
| *Missing* | *1 (.5%)* | *32 (8%)* | *--* |
| Primary caregiver’s education | | |  |
| *High school or less* | 17 (9%) | 36 (9%) | 1.08 |
| *Some post-secondary education* | 77 (41%) | 170 (40%) | 1.04 |
| *Bachelor’s degree or higher* | 90 (48%) | 187 (44%) | 1.17 |
| *Missing* | *(2%)* | *28 (7%)* | *--* |
| Primary caregiver’s ethnicity | | |  |
| *White* | 138 (74%) | 286 (68%) | 1.36 |
| *Other* | 45 (24%) | 102 (24%) | 1.00 |
| *Missing* | *3 (2%)* | *33 (8%)* | *--* |

Note. Odds ratio test results are all non-significant (*p* > .05). ^†^Demographic information was collected at study entry (2-5 years old).

Table S2. Internal consistency of PEM-CY (Cronbach’s α)

|  | T1 | T2 |
| --- | --- | --- |
| Frequency | | |
| Home | .53 | .63 |
| School | .66 | .52 |
| Community | .68 | .66 |
| Involvement | | |
| Home | .77 | .79 |
| School | .81 | .76 |
| Community | .87 | .86 |
| Environmental Support | | |
| Home | .80 | .81 |
| School | .82 | .84 |
| Community | .84 | .85 |

Table S3. Descriptive statistics of all numeric variables

Latent profile indicators (PEM-CY frequency, excluding outliers)

|  | T1 | | | | | T2 | | | | |
| --- | --- | --- | --- | --- | --- | --- | --- | --- | --- | --- |
|  | **N** | **Mean** | **SD** | **Skew.** | **Kurt.** | **N** | **Mean** | **SD** | **Skew.** | **Kurt.** |
| Home | | | | | | | | | | |
| Computer games | 153 | 6.43 | 1.62 | -3.22 | 9.26 | 128 | 6.37 | 1.49 | -3.01 | 8.91 |
| Indoor play | 150 | 4.63 | 2.30 | -.78 | -.70 | 128 | 3.59 | 2.39 | -.19 | -1.32 |
| Arts/hobbies | 152 | 5.57 | 2.03 | -1.68 | 1.77 | 128 | 5.41 | 2.13 | -1.33 | .56 |
| Watching TV | 152 | 6.57 | .92 | -3.86 | 20.16 | 128 | 6.58 | .83 | -2.39 | 5.86 |
| Together w/ people | 153 | 5.61 | 1.50 | -1.22 | 1.15 | 128 | 5.35 | 1.76 | -.95 | -.11 |
| Social w/ tech | 153 | 4.44 | 2.87 | -.65 | -1.32 | 127 | 4.54 | 2.83 | -.65 | -1.31 |
| House chores | 154 | 5.42 | 1.96 | -1.68 | 1.98 | 128 | 5.43 | 1.77 | -1.63 | 2.33 |
| Personal care | 154 | 6.95 | .21 | -4.32 | 16.79 | 128 | 6.84 | .58 | -4.28 | 19.79 |
| School preparation | 153 | 5.04 | 2.70 | -1.09 | -.49 | 128 | 5.54 | 2.50 | -1.55 | .69 |
| Homework | 153 | 5.03 | 2.62 | -1.15 | -.33 | 128 | 4.88 | 2.69 | -1.03 | -.66 |
| School | | | | | | | | | | |
| Classroom | 155 | 6.04 | 1.96 | -2.36 | 4.35 | 132 | 6.30 | 1.67 | -2.95 | 7.81 |
| Field trips/events | 154 | 2.82 | 1.67 | .41 | -.02 | 131 | 2.84 | 1.72 | .62 | -.15 |
| School clubs | 154 | 2.04 | 2.60 | .68 | -1.30 | 132 | 2.02 | 2.65 | .69 | -1.34 |
| Together w/ peers | 154 | 3.54 | 3.04 | -.07 | -1.79 | 132 | 3.60 | 2.91 | -.11 | -1.69 |
| Special roles | 154 | 1.50 | 2.46 | 1.31 | .08 | 130 | 1.67 | 2.53 | 1.15 | -.35 |
| Community | | | | | | | | | | |
| Neighbourhood outings | 155 | 5.17 | 1.22 | -1.26 | 2.05 | 130 | 4.92 | 1.54 | -1.16 | .84 |
| Community events | 154 | 1.99 | 1.46 | .52 | -.18 | 130 | 1.72 | 1.55 | .53 | -.65 |
| Organized physical | 154 | 3.24 | 2.58 | -.18 | -1.63 | 129 | 2.72 | 2.56 | .15 | -1.69 |
| Unstructured physical | 155 | 4.06 | 1.98 | -.60 | -.50 | 130 | 3.45 | 2.25 | -.28 | -1.27 |
| Lessons | 154 | 2.08 | 2.44 | .55 | -1.47 | 130 | 1.83 | 2.54 | .87 | -1.00 |
| Group/volunteer | 157 | 1.01 | 1.91 | 1.53 | .61 | 131 | 1.10 | 1.99 | 1.46 | .40 |
| Religious | 157 | 1.31 | 2.02 | 1.22 | -.14 | 131 | 1.56 | 2.11 | .97 | -.67 |
| Together w/ other kids | 157 | 1.95 | 2.14 | .67 | -.98 | 131 | 1.85 | 2.04 | .59 | -1.20 |
| Working for pay | 157 | .96 | 1.95 | 1.79 | 1.66 | 130 | .98 | 2.04 | 1.86 | 1.93 |
| Overnight trips | 156 | 1.13 | 1.18 | .93 | .28 | 131 | 1.12 | 1.25 | 1.05 | .48 |

Other variables for examining associations with latent profiles

|  | N | | Mean | | | SD |
| --- | --- | --- | --- | --- | --- | --- |
| PEM-CY (composite scores) | | | | | | |
| Involvement Home | 157 | | 3.79 | | | .63 |
| School | 149 | | 3.70 | | | .94 |
| Community | 157 | | 3.71 | | | .82 |
| Environmental support Home | 157 | | .59 | | | .28 |
| School | 156 | | .59 | | | .26 |
| Community | 157 | | .46 | | | .27 |
| Nonverbal IQ |  | |  | | |  |
| WASI-II perceptual reasoning index | 127 | | 95.10 | | | 21.74 |
| Leiter-R/Leiter-3 NVIQ | 22 | | 37.82 | | | 5.76 |
| ADOS (calibrated severity score) | | 152 | | 6.66 | 2.72 | |
| VABS II (domain standard scores) | | | | | | |
| Socialization | 155 | | 70.75 | | | 16.66 |
| Communication | 155 | | 69.77 | | | 15.11 |
| Daily living skills | 155 | | 68.57 | | | 17.69 |
| CBCL 6-18 (domain standard scores) | | | | | | |
| Internalizing behaviour | 158 | | 57.42 | | | 10.06 |
| Externalizing behaviour | 158 | | 52.15 | | | 9.96 |

Table S4. Model fit statistics for latent profile analysis at each assessment

BIC=Bayesian information criterion; SABIC=sample size-adjusted Bayesian information criterion; LMR-LRT=Lo-Mendell-Rubin likelihood ratio test. The fit statistics of the selected solution were bolded.

Note. While model fit statistics and latent profile proportions were the major criteria for model selection, we also considered the same number of profiles over time to facilitate the interpretation of the latent transition analysis (Ryoo et al., 2018).

| **T1** | | | | | |
| --- | --- | --- | --- | --- | --- |
| # of Profiles | BIC | SABIC | LMR-LRT | Entropy | Proportions (%) |
| Home (n=154; excluding 4 outliers) | | | | | |
| 2 | 5425.05 | 5519.20 | .01 | 1.00 | 6/94 |
| **3** | **5341.37** | **5208.37** | **.08** | **.996** | **6/21/73** |
| 4 | 5295.37 | 5127.61 | .41 | .997 | 4/7/20/69 |
| School (n=155) | | | | | |
| 2 | 3285.94 | 3235.30 | <.0001 | 1.00 | 10/90 |
| **3** | **3131.77** | **3062.14** | **.03** | **.990** | **9/35/56** |
| 4 | 3086.01 | 2997.38 | .04 | .988 | 10/12/17/61 |
| 5 | 3008.82 | 2901.20 | .16 | .983 | 9/11/13/23/44 |
| Community (n=157; excluding 1 outlier) | | | | | |
| **2** | **6156.42** | **6058.29** | **.002** | **.999** | **20/80** |
| 3 | 6054.20 | 5921.26 | .04 | .999 | 7/16/77 |
| 4 | 5996.77 | 5829.00 | .21 | .999 | 6/11/14/69 |
| **T2** | | | | | |
| # of Profiles | BIC | SABIC | LMR-LRT | Entropy | Proportions (%) |
| Home (n=128; excluding 5 outliers) | | | | | |
| 2 | 4861.50 | 4763.47 | .001 | 1.00 | 17/83 |
| **3** | **4793.86** | **4661.03** | **.62** | **.998** | **5/21/74** |
| 4 | 4740.99 | 4573.38 | .40 | .996 | 3/14/14/69 |
| School (n=132) | | | | | |
| 2 | 2765.65 | 2715.04 | .0001 | 1.00 | 7/93 |
| **3** | **2574.75** | **2505.17** | **.0002** | **.994** | **7/36/57** |
| 4 | 2538.58 | 2450.02 | .026 | .996 | 7/7/30/55 |
| 5 | 2513.25 | 2405.71 | .56 | .984 | 7/11/13/23/46 |
| Community (n=130) | | | | | |
| **2** | **5298.29** | **5200.24** | **.0007** | **1.00** | **19/81** |
| 3 | 5246.95 | 5114.11 | .15 | .995 | 8/19/73 |
| 4 | 5198.61 | 5030.98 | .24 | .995 | 8/9/11/72 |

Table S5. T1 latent profile description and comparisons

Home

|  | HP1  (n=32) | HP2  (n=10) | HP3  (n=112) | Comparison χ^2^ | |
| --- | --- | --- | --- | --- | --- |
|  |  |  |  | Overall (df=2) | Pairwise (z) |
|  | est. mean (SE) | | |  |  |
| Computer games | 6.94 (.04) | .60 (.25) | 6.81 (.05) | 59.56^***^ | 1>2 (7.4^***^), 3>2 (7.4^***^) |
| Indoor play | 4.37 (.40) | 4.00 (.92) | 4.75 (.21) | 1.38 | -- |
| Arts/hobbies | 4.84 (.45) | 5.30 (.86) | 5.80 (.16) | 2.99 | -- |
| Watching TV | 6.69 (.11) | 6.70 (.15) | 6.53 (.10) | .51 | -- |
| Together w/ people | 5.41 (.30) | 5.40 (.51) | 5.69 (.14) | .72 | -- |
| Social w/ tech | 3.03 (.54) | 2.40 (.92) | 5.03 (.24) | 12.77^***^ | 3>1 (3.0^**^), 3>2 (2.4^*^) |
| House chores | 4.16 (.41) | 4.00 (.79) | 5.91 (.14) | 25.14^***^ | 3>1 (4.6^***^), 3>2 (2.6^*^) |
| Personal care | 6.88 (.06) | 7.00 (.00) | 6.97 (.02) | 6.00 | -- |
| School preparation | .27 (.14) | 5.30 (.75) | 6.40 (.09) | 86.00^***^ | 2>1 (4.2^***^), 3>1 (9.3^***^) |
| Homework | 2.49 (.53) | 4.40 (.96) | 5.83 (.18) | 27.24^***^ | 3>1 (5.2^***^) |
| *Avg. Posterior Probability* | *1.000* | *1.000* | *.999* |  | |

School

|  | SP1  (n=14) | SP2  (n=87) | SP3  (n=54) | Comparison χ^2^ | |
| --- | --- | --- | --- | --- | --- |
|  |  |  |  | Overall (df=2) | Pairwise (z) |
|  | est. mean (SE) | | |  |  |
| Classroom | .35 (.24) | 6.57 (.08) | 6.65 (.11) | 53.12^***^ | 2>1 (6.8^***^), 3>1 (7.1^***^) |
| Field trips/events | 1.65 (.44) | 2.72 (.18) | 3.29 (.21) | 10.94^**^ | 3>1 (3.1^**^), 3>2 (2.2^*^) |
| School clubs | .00 (.00) | .28 (.08) | 5.44 (.14) | 127.53^***^ | 3>1 (6.9^***^), 3>2 (10.8^***^) |
| Together w/ peers | .43 (.30) | 3.00 (.33) | 5.21 (.33) | 32.87^***^ | 2>1 (3.0^**^), 3>1 (5.2^***^), 3>2 (4.1^***^) |
| Special roles | .14 (.14) | 1.51 (.27) | 1.84 (.34) | 6.47^*^ | 3>1 (2.5^*^) |
| *Avg. Posterior Probability* | *.999* | *.995* | *.993* |  | |

|  | CP1  (n=125) | CP2  (n=32) | Comparison χ^2^ |
| --- | --- | --- | --- |
|  | est. mean (SE) | | CP1 vs. 2 (df=1) |
| Neighbourhood outings | 5.09 (.11) | 5.52 (.17) | 3.46 |
| Community events | 1.80 (.12) | 2.74 (.27) | 8.99^**^ (2>1) |
| Organized physical | 3.09 (.23) | 2.84 (.44) | 2.25 |
| Unstructured physical | 3.99 (.18) | 4.36 (.32) | .45 |
| Lessons | 1.89 (.22) | 2.81 (.41) | 5.15^*^ (2>1) |
| Group/volunteer | .09 (.03) | 4.63 (.17) | 132.35^***^ (2>1) |
| Religious | 1.02 (.16) | 2.44 (.42) | 12.41^***^ (2>1) |
| Together w/ other kids | 1.80 (.19) | 2.53 (.40) | 3.25 |
| Working for pay | .77 (.16) | 1.72 (.43) | 5.10^*^ (2>1) |
| Overnight trips | 1.01 (.10) | 1.59 (.21) | 6.95^**^ (2>1) |
| *Avg. Posterior Probability* | *1.000* | *1.000* |  |

Community

Kruskal-Wallis rank-sum tests were performed for overall group comparison followed by Dunn’s test for pairwise comparisons (z-test statistics with Benjamini-Hochberg adjusted *p* values); SE=standard error; ^*^*p* < .05, ^**^*p* < .01, ^***^*p* < .001

|  | # Par’s | LL | BIC | Entropy | Satorra-Bentler Scaled LRT χ^2^(df) |
| --- | --- | --- | --- | --- | --- |
| Home | | | | | |
| 1. LTA, non-invariance | 88 | -4852.76 | 10163.96 | .890 | 1 vs. 2: 78.4(30), *p* < .001  1 vs. 3: 33.76(20), *p* = .03 |
| 2. LTA, full invariance | 58 | -4891.96 | 10086.08 | .879 |  |
| 3. LTA, partial invariance | **68** | **-4869.64** | **10093.53** | **.890** |  |
| School | | | | | |
| 1. LTA, non-invariance | 48 | -2724.41 | 5698.88 | .853 | 1 vs. 2: 8.42 (15), *p* = .91 |
| 2. LTA, full invariance | **33** | **-2728.62** | **5629.16** | **.853** |  |
| Community | | | | | |
| 1. LTA, non-invariance | 63 | -5567.42 | 11464.05 | .862 | 1 vs. 2: 30.16(20), *p* = .07 |
| 2. LTA, full invariance | **43** | **-5582.50** | **11271.83** | **.862** |  |

Table S6. Latent transition analysis (LTA) model comparison

Note. The fit statistics of the selected solution were bolded. LRT=likelihood ratio test

Table S7. Transition patterns of “movers” for each setting among participants who had PEM-CY data at both time-points

Home (N=25)

| T1→T2 profile membership | N | % | Transition patterns in other settings | |
| --- | --- | --- | --- | --- |
|  |  |  | School | Community |
| HP1 → HP3 | 11 | 44 | - 60% stayer  - 7% moved to SP1 | - 87% stayer  - 13% moved to CP1 |
| HP2 → HP3 | 4 | 16 |  |  |
| HP1 → HP2 | 1 | 4 | - 60% stayer  - 30% moved to SP1 | - 100% stayer  - 0% moved to CP1 |
| HP2 → HP1 | 0 | 0 |  |  |
| HP3 → HP1 | 6 | 24 |  |  |
| HP3 → HP2 | 3 | 12 |  |  |

School (N=30)

| T1→T2 profile membership | N | % | Transition patterns in other settings | |
| --- | --- | --- | --- | --- |
|  |  |  | Home | Community |
| SP1 → SP3 | 1 | 3 | - 78% stayer  - 0% moved to HP1 | - 56% stayer  - 22% moved to CP1 |
| SP2 → SP3 | 8 | 27 |  |  |
| SP1 → SP2 | 4 | 13 | - 57% stayer  - 10% moved to HP1 | - 86% stayer  - 14% moved to CP1 |
| SP2 → SP1 | 7 | 23 |  |  |
| SP3 → SP1 | 0 | 0 |  |  |
| SP3 → SP2 | 10 | 33 |  |  |

Community (N=23)

| T1→T2 profile membership | N | % | Transition patterns in other settings | |
| --- | --- | --- | --- | --- |
|  |  |  | Home | School |
| CP1 → CP2 | 10 | 43 | - 100% stayer  - 0% moved to HP1 | - 80% stayer  - 0% moved to SP1 |
| CP2 → CP1 | 13 | 57 | - 85% stayer  - 0% moved to HP1 | - 62% stayer  - 0% moved to SP1 |

Figure S1. Distribution of profile memberships across settings at T1 (ages 11-14)


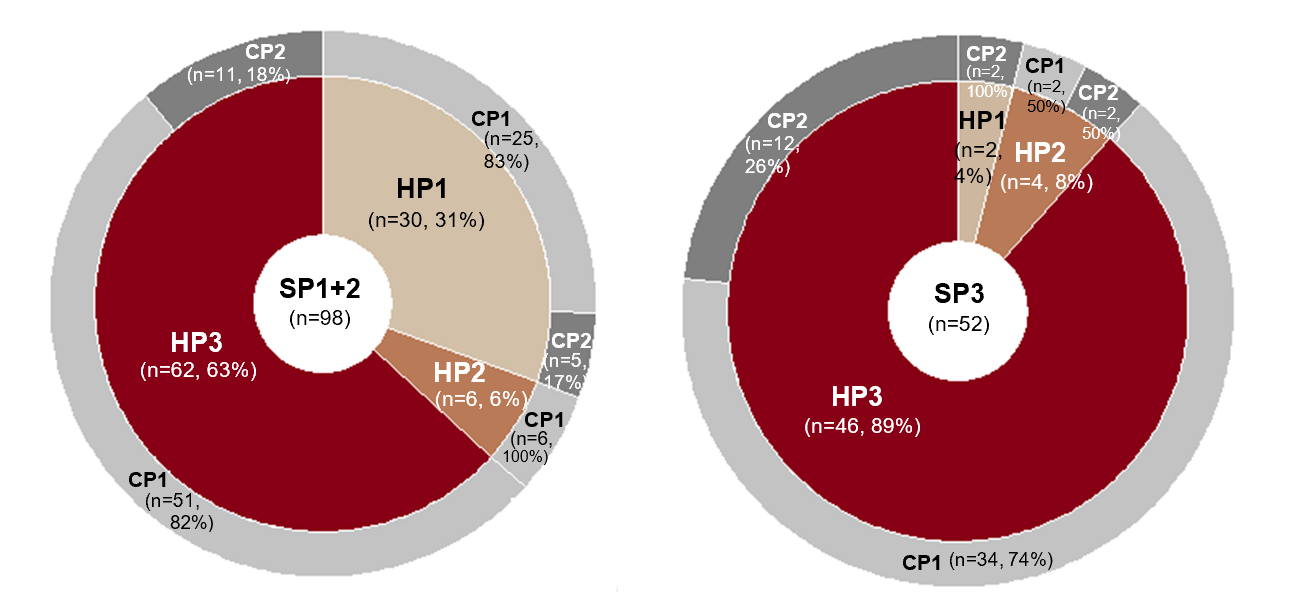


Autistic youth in SP1 and SP2 (the lower school participation profiles) were more likely to be in HP1 characterized by less frequent school preparation at home and CP1 characterized by lower community participation.

Figure S2. Final LTA solutions for each setting


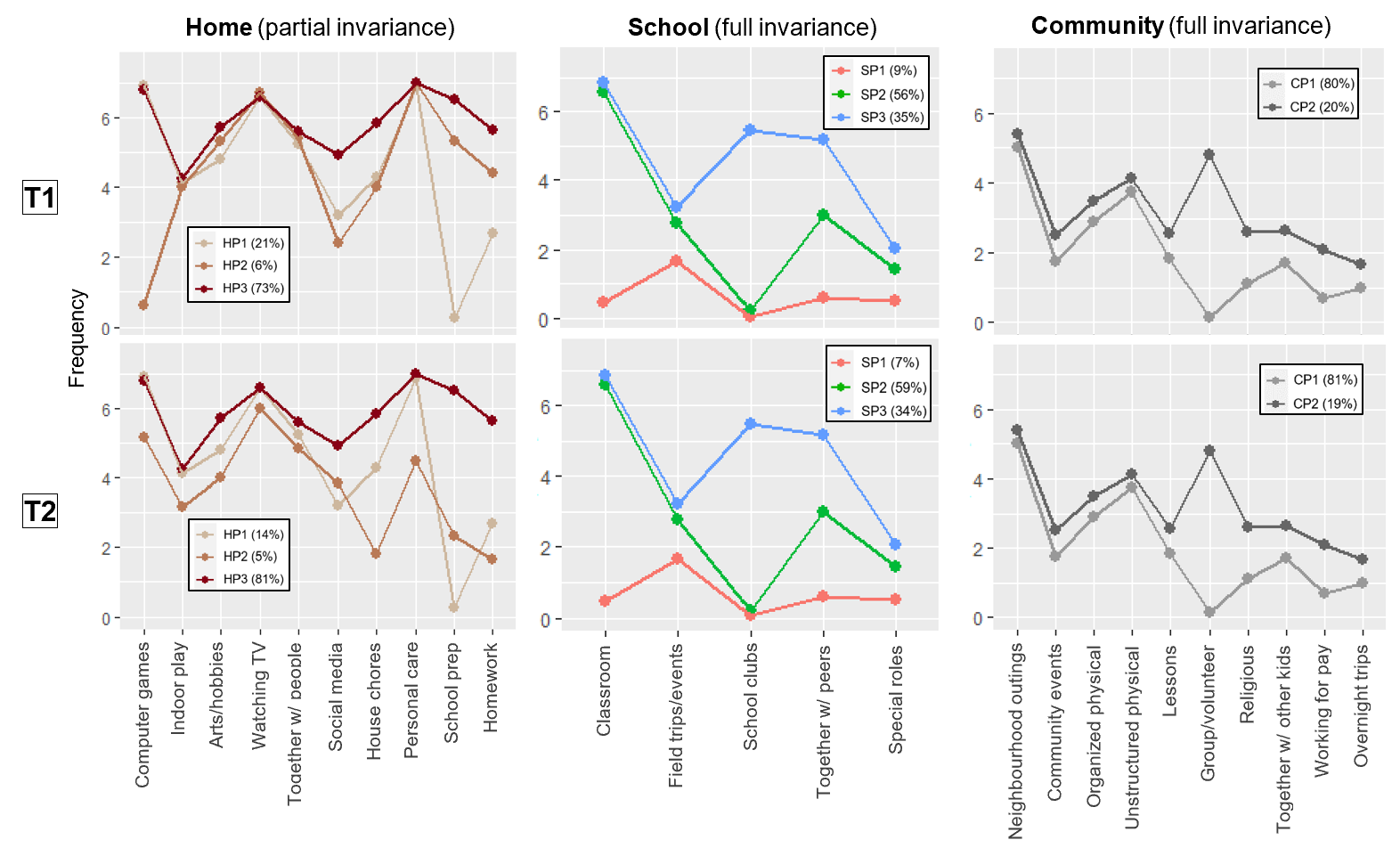


Overnight trips

Working for pay

Together w/ other kids

Religious

Group/volunteer

Lessons

Unstructured physical

Organized physical

Community events

Neighbourhood outings

Special roles

Together w/ peers

School clubs

Field trips/events

Classroom

Homework

School prep

Personal care

House chores

Social w/ tech

Together w/ people

Watching TV

Arts/hobbies

Indoor play

Computer games
